# Slow cortical potentials capture decision processes during temporal discounting

**DOI:** 10.1111/ejn.12108

**Published:** 2012-12-28

**Authors:** Felix Oswald, Uta Sailer

**Affiliations:** 1 Department of Basic Psychological Research and Research Methods, Faculty of Psychology, University of ViennaVienna, Austria; 2 Department of Psychology, University of GothenburgGothenburg, Sweden

**Keywords:** delay discounting, electroencephalography, ERP, human, reward, slow cortical potential (SCP)

## Abstract

Various neuroimaging studies have detected brain regions involved in discounting the value of temporally delayed rewards. This study used slow cortical potentials (SCPs) to elaborate the time course of cognitive processing during temporal discounting. Depending on their strength of discounting, subjects were categorised as low and high impulsive. Low impulsives, but not high impulsives, showed faster reaction times for making decisions when the delayed reward was of high amount than when it was of low amount. Both low impulsives and high impulsives chose the delayed reward more often when its amount was high than when it was low, but this behavior was more pronounced for low impulsives. Moreover, only low impulsives showed more negative SCPs for low than for high amounts. All three measures indicated that only low impulsives experienced extended conflict for delayed low amounts than for high amounts. Additionally, the SCPs of low impulsives were more sensitive to the delay of the delayed reward than those of high impulsives, extending seconds after the response. This indicates that they continued evaluating their choices even after the decision. Altogether, the present study demonstrated that SCPs are sensitive to decision-related resource allocation during inter-temporal decision-making. Resource allocation depended both on the choice situation and on impulsivity. Furthermore, the time course of SCPs suggested that decision-related processes occurred both prior to and after the response.

## Introduction

Decision conflicts between choosing immediate gratification and waiting to obtain an even higher outcome at a later point in time occur permanently in everyday life. For example, one may trade off the gratification of the immediate consumption of unhealthy food against the benefits of not eating it (i.e. a better state of health in the future). However, people are often not willing to wait; they value immediate rewards disproportionately highly (‘present-biased’ choice) (Ainslie, 1975) and discount the value of delayed rewards, a behavior called ‘temporal discounting’ (Samuelson, 1937; Ainslie, 1975).

At a trait level, temporal discounting has been linked to impulsivity (Hinson *et al*., 2003; De Wit *et al*., 2007), and it has also been found to be altered in disorders associated with impulsivity, such as drug addiction (Bickel *et al*., 1999; Kirby *et al*., 1999; Mitchell, 1999; Richards *et al*., 1999; Petry, 2001; Baker *et al*., 2003; Coffey *et al*., 2003), gambling (Petry & Casarella, 1999; Alessi & Petry, 2003), attention deficit hyperactivity disorder (Plichta *et al*., 2009), and antisocial personality disorder (Petry, 2002).

Studies using functional magnetic resonance imaging (fMRI) typically report the ventral striatum, including the nucleus accumbens, the medial prefrontal cortex, the dorsolateral prefrontal cortex (DLPFC), and the posterior cingulate cortex, to be activated during temporal discounting (McClure *et al*., 2004; Kable & Glimcher, 2007; Ballard & Knutson, 2009).

The aim of the current study was to investigate the time course of brain activity during temporal discounting by means of electroencephalography (EEG). We analysed slow cortical potentials (SCPs) in order to capture the temporally extended cognitive processing that takes place when a choice is made between an immediate and a delayed reward.

SCPs are changes in cortical electrical activity that are strongly task-related and last from several hundred milliseconds to several seconds. They are sensitive to a broad spectrum of cognitive processes (Bauer, 1998; Khader *et al*., 2008; He & Raichle, 2009). The amplitude of negative SCPs is correlated with the strength of neural activation as measured with fMRI (Nagai *et al*., 2004; Sabatinelli *et al*., 2007; He *et al*., 2008; Khader *et al*., 2008; He & Raichle, 2009). Thus, similarly to the blood oxygen level-dependent response, SCPs reflect task-related neural activation.

Previous studies have shown that the amount of the delayed reward influences the neural evaluation process of low impulsive subjects more than those of high impulsive subjects (Ballard & Knutson, 2009). Thus, the amount of the delayed reward seems to be particularly relevant for low impulsives. Therefore, relative to low amounts, high amounts should also produce larger changes in SCP amplitude in low impulsives than in high impulsives.

On the basis of the results of Ballard & Knutson (2009), who found higher activation in the DLPFC for short delays, we additionally expected larger SCPs for short delays.

Furthermore, the time course of SCPs in temporal discounting was explored. It was assumed that the time point at which differences in SCP amplitude between the reward conditions would occur may indicate the point at which cognitive resource allocation starts to differ.

## Materials and methods

### Subjects

Thirty right-handed (Oldfield, 1971) students of the University of Vienna (15 males and 15 females, aged between 21 and 35 years) participated in the study after having given informed consent. All of them reported being free of current or past neurological or psychological disorders, and current psychoactive medication.

Of these 30 subjects, 12 were excluded from further analysis, for the following reasons. Six subjects showed a high number of artefacts, which led to the exclusion of too many trials (> 20 trials, leading to < 40 trials per condition). The datasets of two subjects contained a high level of noise in all channels. One subject had reaction times (RTs) below the limit of 700 ms set for stimulus-locked analysis. This limit was chosen because there were only a few trials below 700 ms, and these choices seemed to be very rash and made without much consideration (for an exact definition of EEG epochs that resulted from this criterion, see below). One subject reported that she had not paid attention to the delay information when the delayed reward was high enough. Behavioral data showed that this subject had indeed almost always chosen the delayed reward when its amount was high enough, independent of its delay. All other subjects included in the analysis were influenced by both amount and delay of the delayed reward when making their choices. Two subjects showed high inconsistency in their choice behavior; for example, they were not systematically influenced by reward attributes, or chose the immediate reward in one trial and the delayed reward when the same trial (choice situation) was presented for the second time.

In all, the data from 18 subjects (eight females and 10 males; mean age, 26.6 years [standard deviation (SD), 3.1]) were used for further analysis.

The study was conducted in accordance with the Declaration of Helsinki and local guidelines of the University of Vienna. Written informed consent was obtained from all subjects prior to the study. At the time when the study was carried out, local regulations required no approval of an ethical committee. However, the experiment was supervised and ethically approved by the head of the former Brain Research Laboratory of the Faculty of Psychology, University of Vienna. The study was also performed in the context of a larger project using the same task in an fMRI scanner that was approved by the ethical committee of the Medical University of Vienna.

### Temporal discounting task

Subjects were seated in a dimly lit, sound-attenuated room in front of a table with a 19-inch cathode ray tube monitor (Sony Trinitron Multiscan G520). The distance between the observer's eyes and the monitor was 80 cm. E-Prime 2.0 software (Psychology Software Tools) was used for stimulus presentation and synchronisation during EEG data collection.

In accord with the procedure used by Ballard & Knutson (2009), subjects were asked to choose between a fixed monetary reward of €10, available immediately after the experiment, and a varying higher monetary reward delayed by a few weeks. The immediate reward was held constant to make it possible to attribute possible differences in cortical potential amplitude to specific attributes of the delayed rewards (the amount or the delay). If the immediate reward is constantly €10 and the delayed reward is varied in its amount and delay, differences in SCP amplitude are attributable to variation of the delayed reward only.

**FIG. 1:**
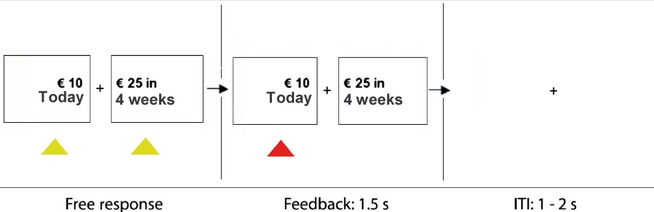
Time course of events within a trial. Left: presentation of the two choice options in boxes: an immediate reward of €10 always on the left side of a fixation cross, and a higher, delayed reward always on the right side. Yellow triangles below the options indicated that a choice was required. Middle: after a choice, in this example of the immediate reward, the triangle below the chosen option turned red. The other one disappeared, in order to show that the response had been successfully logged. Feedback was shown for 1.5 s. Right: only the fixation cross remained on the screen during the variable intertrial interval (ITI) (duration between 1 s and 2 s). Subsequently, the next choice situation appeared on the screen.

In order to obtain a distinct effect, the levels of amounts and delays were chosen in such a way as to ensure equal influence of both on the subjective value (SV) of the delayed reward according to the hyperbolic model:


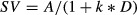


(Mazur, 1987), where *A* is the size of the amount, *D* is the length of the delay (in days), and *k* is an individual discounting parameter. To calculate SV *a priori*, an average *k* of 0.02, as reported in other studies, was assumed (Kable & Glimcher, 2007; Ballard & Knutson, 2009). This procedure resulted in: low amount levels of €11, €12, €13, €14, €15, and €16; high amount levels of €25, €26, €27, €28, €29, and €30; short delay levels of 1, 2, 3, 4 and 5 weeks; and long delay levels of 12, 13, 14, 15 and 16 weeks. Each of the 120 trials was presented twice, so the subjects had to make 240 choices altogether. The task was adapted from McClure *et al*. (2004). The two reward options were presented in boxes (width, 12 cm; height, 6 cm) on either side of the screen, with a fixation cross in the middle (Fig. 1).

The smaller, immediate reward was always presented on the left side of the screen, to avoid eye movements that could occur when the subject is ‘searching’ for the varied reward. Subjects were told to focus on the fixation cross and to avoid eye movements. They were instructed that they were required to choose between two monetary rewards, differing in their amount and delay, according to their preferences. They had as much time as desired to respond by pressing either button 1 (for the immediate reward) or button 3 (for the delayed reward) on the keypad on the right side of the keyboard. Two yellow triangles below the reward options indicated that a choice was required. After a choice, the triangle below the chosen reward turned red for a duration of 1.5 s, to show that the choice had been successfully logged. After that, both reward options disappeared, and only the fixation cross remained on the screen, for a period between 1 s and 2 s. Then, the next trial was presented. The average trial length was 5.1 s.

Every 5 min, a pause of self-determined length occurred to allow the subjects to take a rest. Subjects took approximately 20 minutes to complete the task.

Subjects were told that there were no correct or incorrect answers, and that they should take the time that they needed for every decision. After the experiment, one of their choices would be randomly selected, and they would actually receive the reward they had chosen, either immediately after the experiment if they had chosen the immediate reward, or delayed by the respective weeks (via bank transfer) if they had chosen the delayed reward. To increase the plausibility of this procedure, subjects were asked to fill in their bank details on a piece of paper prior to the experiment.

Following the written instruction, an example of the choice situations was presented on the screen to make the subjects familiar with the task. After that, subjects could initiate the experiment by a button press.

### Barratt Impulsiveness Scale (BIS)

To obtain a personality measure of impulsiveness that can be linked to the strength of temporal discounting, all subjects filled in the German version (Preuss *et al*., 2008) of the BIS (Patton *et al*., 1995) at the end of the experiment. BIS-11, which consists of 30 items, is a widely used self-report scale for assessing impulsiveness.

According to Barratt (Barratt, 1985) and supported by later literature (Stanford *et al*., 2009), impulsiveness is composed of three subtraits: attentional impulsiveness, motor impulsiveness, and non-planning impulsiveness. Attentional impulsiveness concerns attentional deficits and cognitive instability, motor impulsiveness concerns acting without thinking, and non-planning impulsiveness concerns a lack of forethought. In previous studies, subjects with steeper discounting of delayed monetary rewards have been shown to have higher ‘non-planning impulsiveness’ (De Wit *et al*., 2007).

### EEG recording

DC-EEG was performed with 61 cephalic Ag/AgCl ring electrodes, positioned at equal distances in an elastic cap (Easy Cap, montage No. 10; Herrsching, Germany). As electrodes above the preauricular sites (positions FT9 and FT10 in the 10-20 system) were not included in data collection, electrophysiological data were collected from 59 scalp sites.

All cephalic electrodes were referenced to a balanced non-cephalic sterno-vertebral reference (Stephenson & Gibbs, 1951) consisting of two interconnected electrodes, one of which was placed above the right sterno-clavicular junction, and the other above the seventh vertebra. The voltage between these electrodes was balanced with a potential divider (potentiometer) to minimise the visible electrocardiogram amplitude in the EEG channels.

To record the electrooculogram (EOG), four additional electrodes were attached above and below the right eye (for the vertical EOG), and on the outer canthi of both eyes (for the horizontal EOG) (Picton *et al*., 1995). Subsequently, the skin below the electrodes was slightly scratched with a sterile needle, and the electrodes were filled with degassed electrode gel (Electro-Gel; Electrode-Cap International, Eaton, OH, USA). The impedance values of all electrodes were checked with a manual impedance meter to be ≤ 2 kΩ.

The data were sampled at 250 Hz for digital storage. EEG and EOG signals were amplified within a bandwidth from DC to 125 Hz, with a DC amplifier (Ing. Kurt Zickler, Pfaffstätten, Austria).

### Data analysis

#### Behavioral analysis

RT was defined as the interval between stimulus onset and button press, reflecting the time that participants needed for reward evaluation and decision-making (forming and making a choice). RT was computed for each condition (low short, low long, high short, and high long), based on all trials with an RT of > 700 ms per condition, including those coded as artefactual in the EEG analysis. This inclusion of artefactual trials in the RT computation was considered to be acceptable, as the number of artefactual trials was similar among conditions.

Impulsive choice behavior was defined as the frequency of immediate choices as compared with all choices (ratio of impulsive choices). The impulsive choice ratio (ICR) represents a commonly used index of the individual strength of discounting (e.g. Mitchell *et al*., 2005). Subjects were divided into high and low impulsives according to this index, via a median split. To validate this measure of impulsivity, BIS-11 sum score and subscores were correlated with strength of discounting (mean ICR) for each individual and condition by computing Pearson correlations. Furthermore, weakly and strongly discounting subjects were compared regarding their BIS-11 sum score and subscores by use of *t*-tests for independent samples.

Mean ICR was computed separately for low impulsives and high impulsives (i.e. weak and strong discounting) and the following conditions: low short, €10 today vs. €11–16 delayed by 1–5 weeks; low long, €10 today vs. €11–16 delayed by 12–16 weeks; high short, €10 today vs. €25–30 delayed by 1–5 weeks; and high long, €10 today vs. €25–30 delayed by 12–16 weeks.

Mean ICR was then subjected to a repeated-measures anova with amount (low, high) and delay (short, long) as within-subject factor, and impulsivity (low, high, i.e. weak and strong discounting) as between-subjects factor.

In order to analyse RT, a similar repeated-measures anova was conducted with amount (low, high) and delay (short, long) as within-subject factors, and impulsivity (low, high, i.e. weak and strong discounting) as between-subjects factor.

Furthermore, all BIS-11 scales were correlated (Pearson correlations) with mean RTs per subject across conditions, and with RTs per subject and condition, to determine whether there was a relationship between trait impulsiveness and the time that subjects took to make a decision.

#### EEG data processing

Off-line, the data were corrected for eye movement-related artefacts (Bauer & Lauber, 1979; Lamm *et al*., 2005). For further analysis, eeglab 6.03b (Delorme & Makeig, 2004), which is matlab-based (v. 7.5.0; The MathWorks) software, was employed. The data were then low-pass-filtered with a cutoff frequency of 30 Hz (roll-off, 6 dB/octave). Trials with amplitudes exceeding ± 95 μV and a linear trend ≥ 50 μV were rejected following additional visual inspection.

As the mean RT of all trials across subjects was 2093 ms (time between stimulus onset and response), EEG epochs of length 2200 ms, time-locked to stimulus onset, were extracted for each trial. Each trial was then baseline-corrected by subtracting the mean voltage from the 200 ms prior to stimulus onset from the entire trial. Trials with RTs below 700 ms (RT criterion) were excluded to ensure that every EEG epoch of 2200 ms consisted of sufficient time until response and feedback. For example, a trial with the minimum RT of 700 ms would lead to an epoch consisting of 700 ms (time until response) and 1500-ms feedback of the chosen reward. The RT criterion of exactly 700 ms was set because most of the RTs were above this limit and those below were thought to be rash choices made without much consideration. As the mean RT was 2093 ms, the main part of the analysed epoch covered decision processes.

The remaining trials were averaged separately for each subject and condition. The average numbers of valid trials were 52.5 (SD, 4.9) for condition low short, 51.8 (SD, 4.6) for condition low long, 52.1 (SD, 5.2) for condition high short, and 52.8 (SD, 4.5) for condition high long.

As visual inspection of the grand average waveforms of each condition showed similar potentials until 700 ms post-stimulus, SCPs were analysed in the time window between 700 and 2200 ms post-stimulus. Mean amplitudes at electrodes Fz, Cz and Pz were extracted for the time periods 700–1200 ms, 1200–1700 ms, and 1700–2200 ms, respectively, as visual inspection of the SCP time courses suggested such a distinction.

Fz and Cz were selected because SCP amplitudes proved to be maximal above frontal midline sites in other paradigms that caused response conflict (Diener *et al*., 2010). Pz was also selected to account for event-related potential (ERP) differences from anterior to posterior along the midline axis.

To disentangle cognitive processes occurring prior to and after the response, EEG data were also averaged time-locked to the button press. Response-locked data were subjected to the same preprocessing and artefact correction procedures as used for the stimulus-locked data (see above). The same pre-stimulus interval of 200 ms as used in the stimulus-locked analysis served as baseline in the response-locked analysis.

Then, the averaged amplitude of each subject and condition was extracted at each electrode Fz, Cz and Pz for the following time periods: −700 to −200 ms (time interval immediately prior to response, starting at −700 ms, because the fastest response included in the analysis was set to be above 700 ms), 500–1000 ms, and 1000–1500 ms. The time epoch between −200 ms and 500 ms was not analysed, because a response-related potential is visible within this time epoch (Fig. 5). The end of the last time interval was 1500 ms, as that was the end of the trial. The mean amplitude in the first interval (−700 to −200 ms) was supposed to cover pre-response cognitive processes. Mean amplitudes in the last two intervals, between 500 and 1500 ms, were supposed to cover post-response evaluation processes.

#### Analysis of ERPs

Stimulus-locked SCP amplitudes were subjected to a repeated-measures anova with amount (low, high), delay (short, long), time frame (TF) (TF1, 700–1200 ms; TF2, 1200–1700 ms; TF3, 1700–2200 ms) and electrode (Fz, Cz, Pz) as within-subject factors, and impulsivity (low, high, i.e. weak and strong discounting) as between-subjects factor.

As it turned out that high rewards led to a decrease in SCP amplitude (although only with a tendency) in low impulsives, and in a similar fashion to a decrease in RTs, follow-up analyses were performed. The reason was that significantly different RTs between the conditions led to an overlap of cognitive processes occurring before and after the response when stimulus-locked SCPs were compared between conditions. For example, when we compared the stimulus-locked SCPs in the time window between 700 and 2200 ms in the condition ‘high amount, short delay’ and the condition ‘high amount, long delay’ (mean RTs of 1441 and 1565 ms) with the SCPs in ‘low amount, short delay’ and ‘low amount, long delay’ conditions (mean RTs of 1928 and 2160 ms), pre-response-processes (for ‘low amount, short delay’ and ‘low amount, long delay’) partially overlap with post-response processes (for ‘high amount, short delay’ and ‘high amount, long delay’). The stimulus-locked analysis therefore provided a comparison of cognitive processes between conditions irrespective of whether they occurred prior to or after the response.

In contrast, a response-locked analysis allows the separation of pre-response and post-response processes. Moreover, as the ERPs are aligned to the response, potential response-related processes should become evident. Therefore, differences in the stimulus-locked analysis that would hold true in the response-locked analysis both prior to and after the response should be attributable to reward-evaluation processes and not to response-related processes.

Response-locked SCP data were subjected to a repeated-measures anova to analyse pre-response and post-response evaluation processes, with amount (low, high), delay (short, long), TF (TF1, −700 to −200 ms; TF2, 500–1000 ms; TF3, 1000–1500 ms) and electrode (Fz, Cz, Pz) as within-subject factors, and impulsivity (low, high, i.e. weak and strong discounting) as between-subjects factor.

For all anovas, the degrees of freedom (d.f.) associated with effects involving factors with more than two levels were corrected for non-sphericity with the Greenhouse–Geisser procedure, when appropriate. *Post hoc* Tukey HSD tests were performed to explore interaction effects. To demonstrate the effect size of the experimental manipulation, partial eta-squared (*η*^2^) is reported (Cohen, 1973). Partial eta-squared indicates the amount of variance explained by the measured values at the level of the sample (not the population).

To assess whether our analyses had a fair chance of rejecting an incorrect null hypothesis, *post hoc* power analyses were performed with G*Power 3 (Faul *et al*., 2007). We assumed that main effects and interactions of medium size (*f* = 0.25; according to Cohen's effect size conventions) (Cohen, 1988) should be detected, given a significance level of α = 0.05.

To gain insights into the relationship between SCP amplitude and RT, Pearson correlations were computed for RTs in all conditions (low short, low long, high short, and high long) and for SCP amplitude in the corresponding conditions, separately for Fz, Cz, and Pz.

## Results

### Behavioral data

#### RT

The average RT was 2093 ms (SD, 1201 ms): 1774 ms for low impulsives (SD, 634 ms), and 2412 ms (SD, 1562 ms) for high impulsives. The large group difference is attributable to one individual in the high impulsive group who had an average RT of 6130 ms. This subject was included in the analysis, as performing all of the analyses without this subject did not change the results of subsequent analyses (including their effect sizes). Thus, keeping these data did not distort the overall results in a systematic way. In contrast, the elimination of this subject would have decreased the signal-to-noise ratio of the electrophysiological data.

**FIG. 2:**
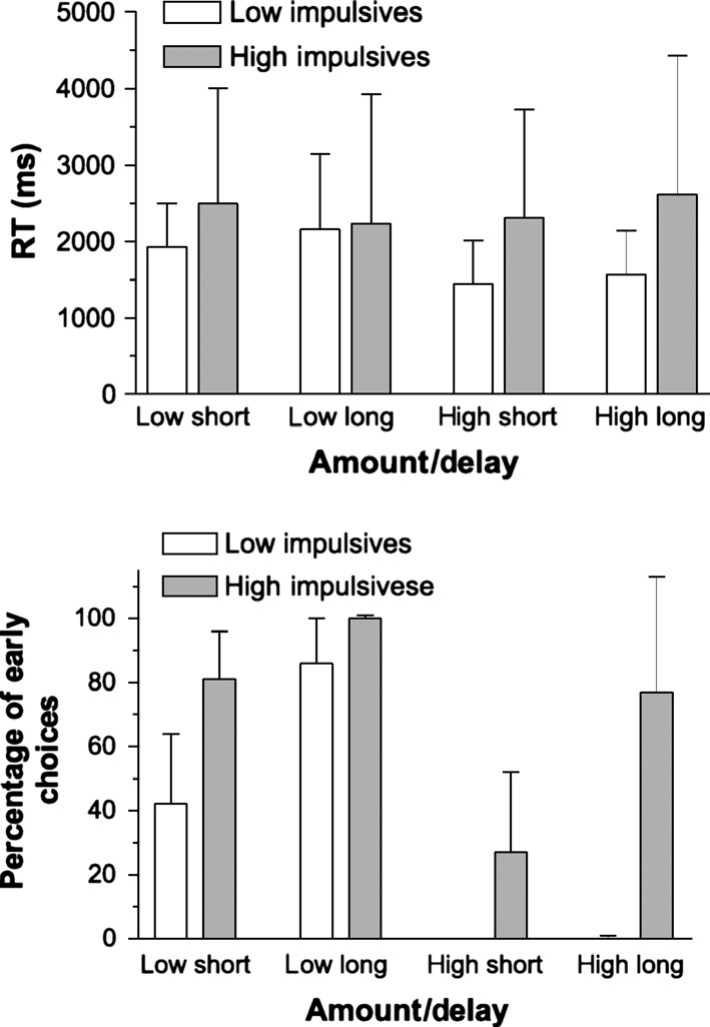
Mean RTs and mean percentage of early choices for the conditions low short, low long, high short, and high long, separately for low impulsives and high impulsives. Error bars indicate standard errors of the mean.

Repeated-measures anova on RTs revealed a significant main effect of amount (*F*_1,16_ = 6.50, *P* < 0.05, *η*^2^ = 0.29), with faster RTs when the amount of the delayed reward was higher. Moreover, both groups differed according to their RTs, as indicated by a significant amount × impulsivity interaction (*F*_1,16_ = 13.63, *P* < 0.01, *η*^2^ = 0.46). Low impulsives showed significantly faster RTs when confronted with high amounts than when confronted with low amounts (*P* < 0.05), which was not the case for high impulsives (Fig. 2).

#### Choice behavior

In 17–87% of all choices per subject, the immediate reward was chosen, with a mean of 47% and a median of 37% across all choice situations (trials) and subjects. Subjects who had chosen the immediate reward in < 37% of all choice situations were assigned to the low impulsive group (four females and five males; mean age, 26.9 years), and subjects who had chosen the immediate reward in > 37% of all choice situations were assigned to the high impulsive group (four females and five males; mean age, 26.3 years). Low impulsives chose the immediate reward with an average of 28%, and high impulsives with an average of 67%.

Repeated-measures anova on the relative frequency of impulsive choices revealed a highly significant main effect of amount (*F*_1,16_ = 99.85, *P* < 0.001, *η*^2^ = 0.86), with more impulsive choices when the amount of the delayed reward was low, a highly significant main effect of delay (*F*_1,16_ = 83.93, *P* < 0.001, *η*^2^ = 0.84), with more impulsive choices when the delay of the delayed reward was long, and a highly significant main effect of impulsivity (*F*_1,16_ = 40.26, *P* < 0.001, *η*^2^ = 0.72).

Furthermore, there was a significant amount × impulsivity interaction (*F*_1,16_ = 5.92, *P* < 0.05, *η*^2^ = 0.27). Both groups chose the immediate reward more often when the amount of the delayed reward was low than when it was high (*P* < 0.001 for low impulsives and *P* < 0.01 for high impulsives). Both groups also chose the immediate reward similarly often when confronted with low, delayed amounts. However, when the delayed amount was high, low impulsives chose the immediate reward less often than high impulsives (*P* < 0.01). In fact, low impulsives almost never chose the immediate reward when the amount of the delayed reward was high. This means that low impulsives' choice behavior was more sensitive to the amount of the delayed reward than high impulsives' choice behavior.

A highly significant amount × delay × impulsivity interaction (*F*_1,16_ = 58.01, *P* < 0.001, *η*^2^ = 0.78) further specified the two-way interaction. The choice behavior of low and high impulsives for high amounts differed, particularly with long delays (Fig. 2). In this case, low impulsives chose the immediate reward less often than high impulsives (*P* < 0.001). Thus, high amounts at long delays were more attractive for low impulsives than for high impulsives.

For the analyses of RT and choice responses, *post hoc* power analysis calculated a power value of 0.24 for the between-subjects main effect (critical *F*_1,16_ = 4.49), and a power value of 0.67 (critical *F*_1,16_ = 2.79) for within-subjects effects and the interaction effect. Thus, the missing between-subjects main effect for RT may be attributable to a lack of statistical power. *Post hoc* power analysis revealed that, on the basis of the observed between-subjects effect size (*f* = 0.28), a sample size of at least 61 individuals would be required in order to obtain statistical power at the recommended 0.80 level (Cohen, 1988). However, even if the analysis may have failed to detect a general difference in RT between low impulsives and high impulsives, the existing interaction points to the fact that the RTs of low impulsives were modulated to a larger extent by the amount of the delayed reward than the RTs of high impulsives.

#### Trait impulsiveness and temporal discounting

Both the BIS-11 sum score and the subscore for non-planning impulsiveness correlated significantly with the ratio of early choices, i.e. the strength of temporal discounting (sum score, *r* = 0.47, *P* < 0.05; non-planning subscore, *r* = 0.48, *P* < 0.05). Thus, subjects showing higher non-planning impulsiveness and higher general impulsiveness also showed stronger discounting of delayed monetary rewards. These results are in line with previous studies (Hinson *et al*., 2003; De Wit *et al*., 2007), and strengthen the validity of using the ratio of early choices as a measure for impulsivity. There was also a tendency for a significant correlation between motor impulsiveness and temporal discounting (*r* = 0.47, *P* = 0.051).

Subjects assigned to the strongly discounting group (high impulsives) by median split showed a tendency to have higher non-planning impulsiveness (*t* = −1.85, d.f. = 16, *P* = 0.082), higher motor impulsiveness (*t* = −2.15, d.f. = 12.26, *P* = 0.052) and higher general impulsiveness (*t* = −2.05, d.f. = 16, *P* = 0.057) than subjects in the weakly discounting group (low impulsives). Because of the strong association between discounting and impulsiveness, using the BIS-11 score as the between-subjects variable instead of discounting strength did not lead to substantially different results in the ERP analysis of individual differences, which are therefore not reported. All correlations between BIS-11 scales and RTs were non-significant.

### ERP data

**FIG. 3:**
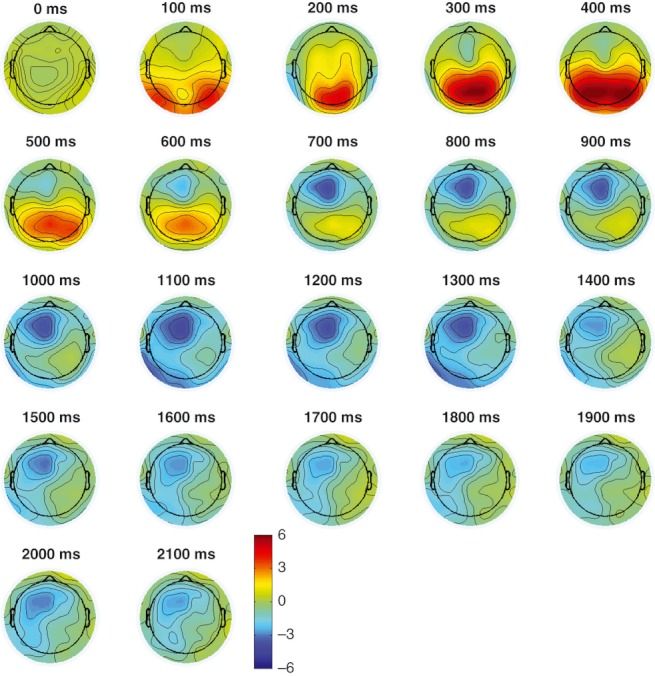
Scalp distribution of SCP amplitude in the condition high short (the distribution did not not vary substantially among conditions). Note the frontal negative maximum, beginning at approximately 700 ms post-stimulus.

**FIG. 4:**
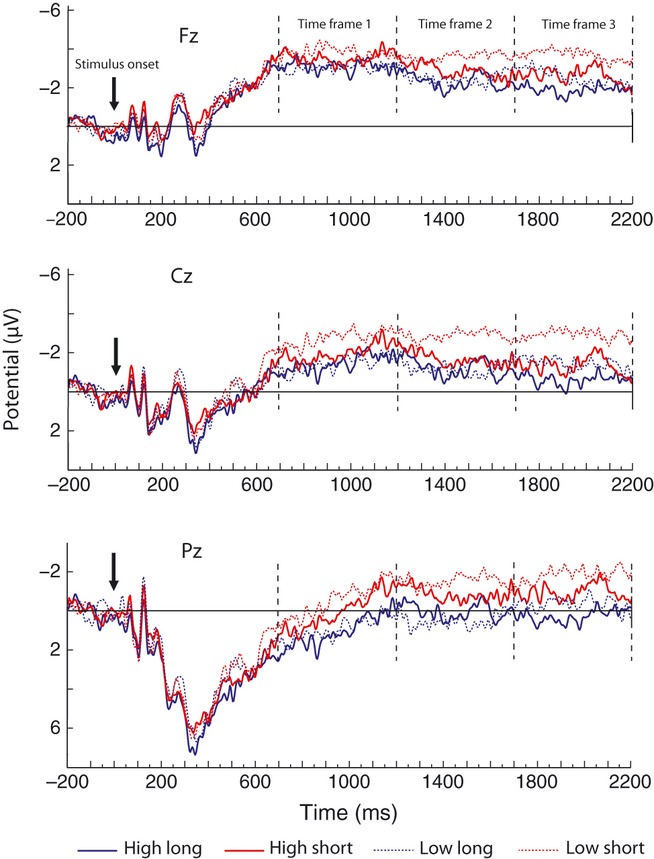
Stimulus-locked grand average waveforms for each condition (thick blue line, high long; thick red line, high short; dotted thin blue line, low long; dotted thin red line, low short) at electrode positions Fz, Cz, and Pz. Negative is plotted upwards. Note the more negative potential above frontal electrode sites (Fz) than above posterior electrode sites (Pz). The divergence of the waveforms for the different conditions is visible at approximately 700 ms.

**FIG. 5:**
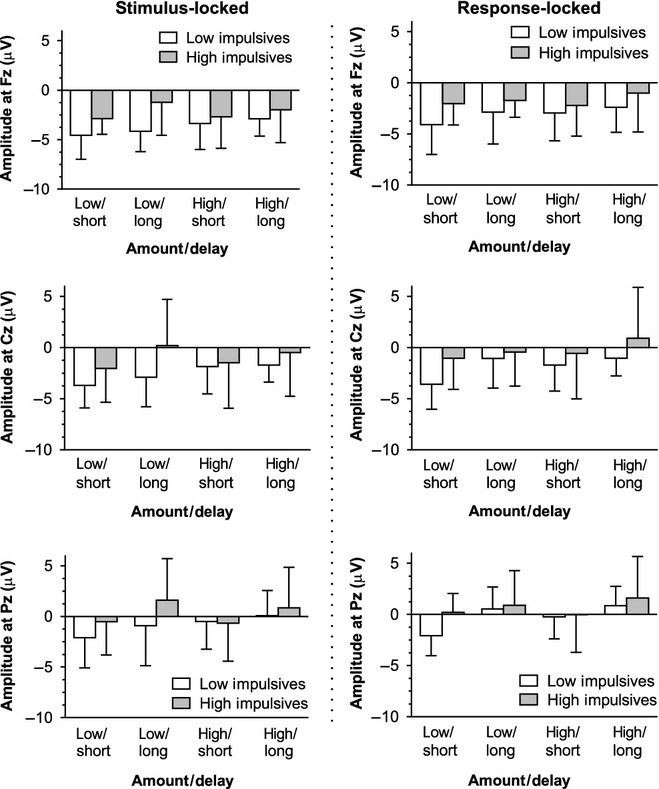
Mean SCP amplitude values (left, stimulus-locked; right, response-locked) for different conditions and impulsivity groups, separately for electrodes Fz, Cz, and Pz. Error bars indicate one SD.

**FIG. 6:**
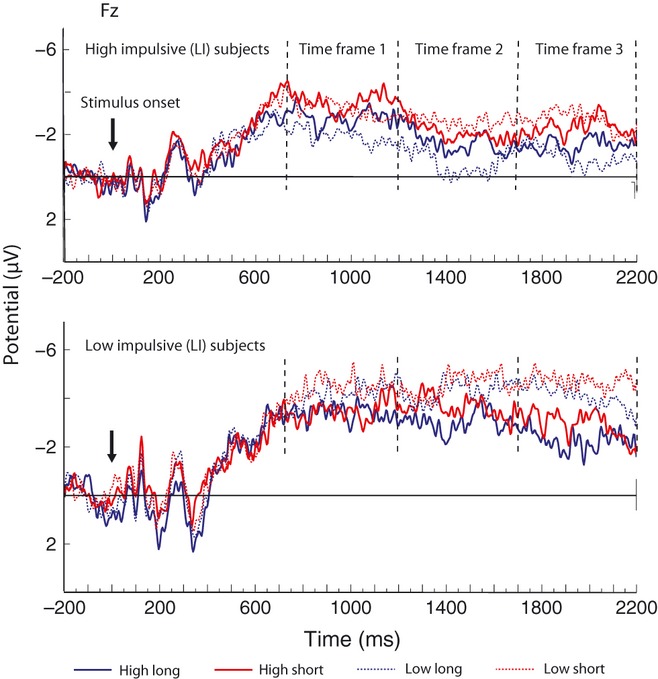
Stimulus-locked grand average waveforms for each condition (thick blue line, high long; thick red line, high short; dotted thin blue line, low long; dotted thin red line, low short) at electrode position Fz, separately for low and high impulsives. Negative is plotted upwards.

Figure 3 shows the scalp topographies of the electrophysiological responses to the choice options. Figure 4 shows stimulus-locked grand average waveforms for the conditions low short, low long, high short and high long separately at Fz, Cz and Pz. Potential amplitudes for the different conditions appeared to be similar up to 700 ms post-stimulus (Fig. 4). However, at approximately 700 ms following stimulus presentation, differences in potential amplitudes, depending on reward attributes, become apparent in the grand averages. These differences lasted for at least 1500 ms. Figure 5 shows means and SDs of stimulus-locked and response-locked SCP amplitudes for all conditions and impulsivity groups, separately for Fz, Cz, and Pz. As is evident from Fig. 4, the amplitudes were generally more negative above frontal electrode sites (Fz) than above posterior sites (Pz). This is in accordance with other studies (e.g. Diener *et al*., 2010). Figure 6 shows grand average waveforms at Fz for the same conditions, separately for low impulsives and high impulsives.

Repeated-measures anova on stimulus-locked SCP amplitudes revealed a significant main effect of delay (*F*_1,16_ = 7.28, *P* < 0.05, *η*^2^ = 0.31). SCPs were more negative for short than for long delays at all measured electrodes. An additional amount × impulsivity interaction (*F*_1,16_ = 5.92, *P* < 0.05, *η*^2^ = 0.27) showed that the effect of amount on SCPs was greater for low impulsives than for high impulsives. Although the difference did not reach significance in the *post hoc* tests, low impulsives showed more negative SCPs for alternatives with low amounts than for those with high amounts. In contrast, the SCPs of high impulsives did not differ with varying amounts.

There was also a main effect of electrode (*F*_2,32_ = 11.29, *P* < 0.001, *η*^2^ = 0.41), and an electrode × TF interaction (*F*_1.4,23.1_ = 11.65, *P* < 0.01, *η*^2^ = 0.42). SCPs in TF1 were more negative at Fz than at Cz (*P* < 0.05) and more negative at Cz than at Pz (*P* < 0.001). SCPs in TF2 were more negative at Fz than at Pz (*P* < 0.001), and showed a tendency to be more negative at Cz than at Pz (*P* = 0.06). There were no significant differences between Fz and Cz. In TF3, SCPs were more negative at Fz than at Pz (*P* < 0.001). There were no significant differences between Fz and Cz or between Cz and Pz. SPCs did not significantly differ across TFs at Fz, Cz, or Pz; that is, there was no significant change in neural activation between 700 and 2200 ms.

For the anova on stimulus-locked SCPs, *post hoc* power analysis calculated a power value of 0.28 for the between-subjects main effect (critical *F*_1,16_ = 4.49), and a power value of 0.99 (critical *F*_1,16_ = 1.44) for within-subjects effects and the interaction. Thus, the power to detect a between-subjects main effect was low. The fact that we did not observe a general difference in SCPs between high impulsives and low impulsives may therefore be attributable to limited statistical power because of the modest sample size (*N* = 18). *Post hoc* power analysis revealed that, on the basis of the observed between-subjects effect size (*f* = 0.38), a sample size of approximately 30 would be required in order to obtain statistical power at the recommended 0.80 level (Cohen, 1988). Although the power to detect a general difference in SCPs between high impulsives and low impulsives was low, the observed interaction showed that the amount of the delayed reward had a larger impact on the SCPs of low impulsives than on those of high impulsives. This interaction is also what was predicted on the basis of our hypothesis. Correlations between RTs and SCP amplitude were not significant.

**FIG. 7:**
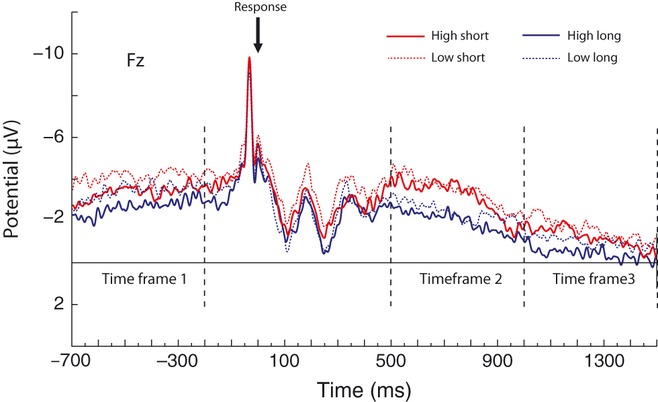
Response-locked grand average waveforms for each condition (thick blue line, high long; thick red line, high short; dotted thin blue line, low long; dotted thin red line, low short) at electrode position Fz. Negative is plotted upwards. In TF1, the interval from −700 to −200 ms, a clear difference between amplitudes for low short and high long is visible, whereas amplitudes for low long and high short seem to be very similar. The difference in amplitude according to delay is best visible in TF2 (+ 500 to + 1000 ms).

Figure 7 shows the response-locked grand average waveforms at Fz separately for the conditions low short, low long, high short, and high long. Repeated-measures anova on the response-locked SCP amplitudes revealed a significant main effect of delay (*F*_1,16_ = 8.18, *P* < 0.05, *η*^2^ = 0.34). As in the stimulus-locked analysis, SCPs were more negative for short delays than for long delays at all measured electrodes. There was also a main effect of electrode (*F*_2,32_ = 12.59, *P* < 0.001, *η*^2^ = 0.44) and a TF × electrode interaction (*F*_1.3,21.2_ = 6.04, *P* < 0.05, *η*^2^ = 0.27).

This interaction was further specified by a delay × electrode × TF × impulsivity interaction (*F*_2.7,43.8_ = 4.33, *P* < 0.05, *η*^2^ = 0.21). For low impulsives, SCP amplitudes were more negative for short than for long delays during all three TFs at both Cz and Pz (all *P*-values < 0.0001), and also at Fz, although this was only a tendency in TF1 (*P* = 0.06 in TF1; *P* < 0.01 in TF2; and *P* < 0.05 in TF3).

For high impulsives, SCP amplitudes were more negative for short than for long delays at Fz during TF2 (*P* < 0.0001), and at Cz and Pz during both TF1 (*P* < 0.05 for Cz, and *P* < 0.0001 for Pz) and TF2 (*P* < 0.0001 for both). Thus, in contrast to low impulsives, high impulsives had similar SCP amplitudes for short and long delays in TF3, i.e. between 1000 ms and 1500 ms after the response.

The amount × impulsivity interaction found in the stimulus-locked analysis was non-significant in the response-locked analysis. However, there was a marginally significant interaction between TF, amount, electrode, and impulsivity (*F*_2.4,38.4_ = 2.92, *P* = 0.06, *η*^2^ = 0.15). Low impulsives showed more negative SCP amplitudes for low than for high amounts during TF1, i.e. 200–700 ms before the response, at all three electrodes (all *P*-values < 0.0001). In contrast, high impulsives only showed this pattern at electrode Cz during TF3 (*P* < 0.0001), i.e. after the response.

## Discussion

The present study addressed the time course of cognitive processing during temporal discounting. We identified slow-wave differences at fronto-central sites for the amount and delay of the later reward, depending on the subjects' impulsiveness.

### Effects of amount

All of the behavioral and electrophysiological measures suggested that low impulsives distinguished between alternatives of low and high amount, whereas high impulsives did so to a much lesser extent. This finding is in accordance with previous research (Ballard & Knutson, 2009), and confirms our hypothesis.

First, the RTs of low impulsives were longer for alternatives with low amounts than for those with high amounts. The RTs of high impulsives did not show such a difference. This suggests that only low impulsives found choices involving high amounts to be easier than choices involving low amounts. Indecisiveness in temporal discounting as measured by a slowing of RTs has been shown to be positively related to activation in conflict-processing areas such as the anterior cingulate cortex (Pine *et al*., 2009). Presumably, lower conflict leads to a faster decision process associated with lower resource allocation.

Second, although all of the subjects chose the delayed reward more often when its amount was high than when it was low, an alternative of high amount made low impulsives choose the delayed option more often than high impulsives. In fact, when the alternative was of high amount, low impulsives almost never chose the immediate reward, even when the delay was long. Thus, amount had a greater influence on the choice behavior of low impulsives than on the choice behavior of high impulsives. Along these lines, high impulsives have been shown to exhibit less neural sensitivity in the mesolimbic reward circuitry in response to the amount of monetary rewards (Goldstein *et al*., 2007; Ballard & Knutson, 2009). High impulsives have been reported to show less nucleus accumbens activation in response to delayed rewards with high amounts than low impulsives (Ballard & Knutson, 2009), which may reflect their reduced attraction to delayed rewards of high amount.

The differential sensitivity of low and high impulsives to delayed rewards with varying amounts in our study can be even further specified. The higher sensitivity of low impulsives to the amount of delayed rewards was particularly evident when, at the same time, the delay was long. Whereas high impulsives also preferred the delayed, higher amount when the delay was short, a long delay made them choose the immediate reward instead. Rational and patient decision-making is considered to require impulse control, which in turn requires cognitive resources (e.g. Heatherton & Wagner, 2011). Low impulsives, who show weak temporal discounting, acted rationally, and almost always chose the delayed reward when its amount was high. When the delayed amount was low, and particularly when the delay was long as well, low impulsives often chose the immediate reward, as high impulsives did, but their RTs slowed down, indicating high cognitive conflict. This may indicate that the decision involved a larger conflict in low impulsives than in high impulsives.

Third, the stimulus-locked SCPs of low impulsives were more negative for low than for high amounts. The SCPs of high impulsives did not show such a difference. A similar effect was found in the response-locked SCPs of low impulsives prior to the response. Only in low impulsives were the SCPs more negative for low than for high amounts. We propose that the more negative SCPs reflect the larger degree of conflict processing (Diener *et al*., 2010) associated with low amounts or the greater allocation of cognitive resources (Rösler *et al*., 1997; Birbaumer, 1999) that are required to resolve the conflict.

### Effects of delay

All subjects showed more negative SCP amplitudes in both stimulus-locked and response-locked analyses for short than for long delays. This finding may parallel the results of Ballard & Knutson (2009), who found the DLPFC to be more strongly activated when subjects were confronted with short than with long delays. Decisions may demand more cognitive resources in lateral prefrontal areas when a choice is being made between two rewards that are close in time than when a choice is being made between two rewards that are far away in time.

The more patient choice behavior of low impulsives was reflected in their SCPs. Whereas both high and low impulsives showed more negative SCPs for short than for long delays, this larger negativity was both more widespread and more sustained in low impulsives, persisting until 1500 ms after the response. In high impulsives, the increased negativity stopped at approximately 1000 ms after the response. These prolonged SCPs in low impulsives may indicate more extensive processing of the delayed reward and/or an ongoing conflict that has not been immediately resolved by the response. As the RTs of low and high impulsives were independent of the delay, both groups seem to have taken equally long to decide between alternatives involving long vs. short delays. However, low impulsives continued to process delay information for a longer time after the response. It has been previously suggested that stimulus identification and analysis, as well as the computation of decision values and response selection, can continue even after a response has been executed (Rabbitt & Vyas, 1981; Carter *et al*., 1998; Sokol-Hessner *et al*., 2012). Subjects are assumed to continue to acquire information following the response, which would allow them to validate or reverse the response. Similarly, with the use of noisy visual stimuli, it has been shown that subjects accumulate information over time until reaching a decision, and continue to process previously ‘unused’ information following an initial choice (Resulaj *et al*., 2009). The present data suggest that a similar ongoing processing of decision-relevant information following a choice may also have occurred in the present study.

In a computational account, Montague & Berns (2002) proposed that post-response activation during the period of reward anticipation may indicate the integration of reward amount and delay into a common internal currency. This currency would allow continuous evaluation of the upcoming reward, in order to decide whether to stay with the current choice or switch to an alternative choice in the future. According to this notion, the ongoing negative SCPs for alternatives with short as compared with long delays in low impulsives may represent the ongoing evaluation of the more difficult or conflict-inducing option in order to guide future decision-making. One may speculate that phenomena such as regret or post-decision dissonance may arise as a result of this type of processing. In any case, these findings illustrate differences in the time course of processing of high and low impulsives that would not have been possible to determine from an fMRI experiment.

### Limitations and implications for future research

In order to validate the assumption of an increasing negative potential being associated with an increase in decision conflict, it would be interesting to use the distance in SV of the two reward options as a conflict measure. This more sensitive measure of conflict could then be used as a predictor of SCP amplitude. In the present study, the chosen increments of the reward variables amount and delay were not continuous, but had ‘gaps’ in between experimental conditions (€11–16 vs. €25–30; 1–5 weeks vs. 12–16 weeks). Thus, they were not well suited for the calculatation of SV according to reported procedures (Kable & Glimcher, 2007; Ballard & Knutson, 2009).

Nevertheless, the present study has provided new insights into the sensitivity of slow cortical potentials to cognitive processes in temporal discounting. Evidently, there was no simple relationship between SV and SCPs, as SCPs were more negative for low (lower SV) than for high (higher SV) amounts in low impulsives, but more negative for short (higher SV) than for long (lower SV) delays in all subjects. This suggests that SCPs are more sensitive to resource allocation in general than to the SV of a reward.

## Conclusion

The current study has provided insights into the sensitivity of slow cortical potentials to cognitive processes in inter-temporal decision-making. The results suggest that there are important differences between low and high impulsives with regard to their cognitive processing of delayed monetary rewards, which are visible in their electrophysiological response. As in previous studies, SCPs in the current study seem to reflect cognitive processes such as conflict monitoring, exertion of cognitive control, and allocation of cognitive resources to solve a decision conflict.

The time course of SCPs suggested that resource allocation was enhanced in certain choice situations as compared with others, not only before but also after a response had been made, indicating the occurrence of post-response evaluation processes.

These findings support the use of SCPs as an interesting complementary method to fMRI for investigation of higher-order cognitive processes in human decision-making, providing extra insights into the time course of cognitive processes.

## Abbreviations

BIS: Barratt Impulsiveness Scale
d.f.: degrees of freedom
DLPFC: dorsolateral prefrontal cortex
EEG: electroencephalography
EOG: electrooculogram
ERP: event-related potential
fMRI: functional magnetic resonance imaging
ICR: impulsive choice ratio
RT: reaction time
SCP: slow cortical potential
SD: standard deviation
SV: subjective value
TF: time frame
